# Suicidality, function and associated negative life events in an adolescent psychiatric population at 3-year follow-up

**DOI:** 10.1186/s12888-021-03100-w

**Published:** 2021-02-18

**Authors:** Kari Skulstad Gårdvik, Terje Torgersen, Marite Rygg, Stian Lydersen, Marit Sæbø Indredavik

**Affiliations:** 1 Regional Centre for Child and Youth Mental Health and Child Welfare, Department of Mental Health, Faculty of Medicine and Health Sciences, Norwegian University of Science and Technology, Trondheim, Norway; 2 Department of Children and Youth, Division of Mental Health Care, St. Olavs Hospital, Trondheim University Hospital, Trondheim, Norway; 3 Orkdal District Psychiatric Centre, Division of Mental Health Care, St. Olavs hospital, Trondheim University Hospital, Trondheim, Norway; 4 Department of Mental Health, Faculty of Medicine and Health Sciences, Norwegian University of Science and Technology, Trondheim, Norway; 5 Department of Clinical and Molecular Medicine, Faculty of Medicine and Health Sciences, Norwegian University of Science and Technology, Trondheim, Norway; 6 Department of Pediatrics, St. Olavs hospital, Trondheim University Hospital, Trondheim, Norway

**Keywords:** Suicidal ideation, Suicidal behavior, School dropout, Adolescent, Negative life event, Longitudinal study

## Abstract

**Background:**

We aimed to examine psychosocial function, suicidality and school dropout in a clinical psychiatric population over a 3-year period from adolescence to young adulthood and explore associations with negative life events.

**Methods:**

This study is part of the Health Survey in Department of Children and Youth, St. Olavs hospital, Norway. In the first study visit (T_1_), 717 (43.5% of eligible) participated, aged 13–18 years (2009–2011), and 3 years later (T_2_), 570 answered a questionnaire (school functioning and negative life events), and 549 completed Kiddie SADS as telephone interview assessing DSM-IV diagnoses, psychosocial functioning and suicidality.

**Results:**

Suicidal ideation was more frequent among girls (17.9%) than among boys (5.4%) (risk difference; RD = 12.5%, CI (7.2 to 17.7), *p* < 0.001), as was suicidal behavior (25.0% vs. 9.5%, RD = 15.5%, CI (9.2 to 21.4), *p* < 0.001). Girls had lower psychosocial functioning than boys (Children’s Global Assessment Scale; Mean score 68.2 vs. 75.2, Mean difference = − 7.0, CI (− 9.4 to − 4.7), *p* < 0.001), and more school dropout (22.5% vs. 13.2%, RD = 9.3%, CI (2.8 to 15.5), *p* = 0.006). For those with a psychiatric disorder, 24.8% of girls had suicidal ideation and 30.0% suicidal behavior, which was larger than for boys (RD = 18.0%, CI (10.8 to 24.7), *p* < 0.001, and RD = 18.3%, CI (10.2 to 25.8), *p* < 0.001, respectively). Exposure to negative life events was frequent for both genders, but more girls had experienced sexually uncomfortable or abusive situations, the last 3 years (23.5% vs. 2.9%, RD = 20.6%, CI (15.4 to 25.7), *p* < 0.001), and ever (44.4% vs. 7.9%, RD = 36.5%, CI (29.9 to 42.7), *p* < 0.001). Suicidal behavior was associated with having been threatened, physically harassed or violently hurt (RD = 16.7%, CI (9.5 to 23.9), *p* < 0.001), and for girls been put into sexually uncomfortable or abusive situations (RD = 20.1%, CI (10.4 to 29.9), *p* < 0.001) and seen others violently hurt (RD = 14.6%, CI (3.4 to 25.8), *p* = 0.011).

**Conclusions:**

The high frequency of suicidality and school dropout confirms the severity of adolescent psychiatric disorders, especially among girls. Specific life events were associated risk factors and should be target points for prevention and intervention.

**Supplementary Information:**

The online version contains supplementary material available at 10.1186/s12888-021-03100-w.

## Background

Adolescence is the period for transitioning into young adulthood and is usually a time of life characterized by good physical health [1]. However, the majority of mental disorders develop during adolescence and contribute to reduced psychosocial function [1–3]. Suicidal symptoms increase during this developmental period [4–6], with a rapid shift from suicidal ideation to suicidal behavior [5, 7, 8], and an estimated lifetime prevalence of suicidal ideation and suicide attempts of 12.1–33% and 4.1–9.3%, respectively [5, 9]. The prevalence of self-harm, defined broadly regardless of motivation and intention to die, has increased among Norwegian adolescents from 4.1 to 16.2% between 2002 and 2018 [10]. Second to road injury, suicide is the most common cause of death among young people worldwide, uncommon before 15 years of age but the frequency increases through adolescence [6, 11–13]. The prevalence across all ages, countries and gender is 3.77/100000, and in Norway 3.00/100000 [12]. Suicide characteristics differ by gender [6, 12–15], with girls having higher rates of suicidal thoughts and behavior, and boys highest rates of committed suicide. Suicidal ideation and behavior are common in patients with psychiatric disorders [5, 6, 16] and are more than three times more frequent in clinical samples of youth than in the general population [7, 17]. Accordingly, the rise of suicidal thoughts and behavior through adolescence coexists with increasing frequencies of psychiatric disorders and related psychopathology that by itself provide higher suicide risk, as for example depression, substance use and some anxiety disorders [5, 7, 16].

It is common to have experienced negative or stressful life events or adversities from childhood to young adulthood [18, 19]. Many different life events are found to be associated with youth suicidal symptoms [20–22]. Such events may include being exposed or witnessed to violence, sexual trauma, or other injury and trauma [19], which are more frequent in clinical psychiatric samples than in the general population [23, 24]. In a systematic review, young people with attempted suicide were more likely to have experienced stressful life events than those with suicidal ideation [20]. A meta-analysis provided strong evidence that early exposure to any interpersonal violence increased the risk of suicide attempts [25]. Many other negative life events have shown associations to suicidal ideation, behavior or committed suicide, as for example death of a parent or a loved one [26, 27], experiences of disasters or accidents [27], peer victimization [28] and multiple other family factors [29, 30]. Experiencing negative life events during demanding developmental periods in childhood and adolescence may increase vulnerability to mental distress by inducing biological changes with long-term effects on nervous, endocrine and immune systems [20, 31]. Thus, negative life events may increase the risk for psychiatric symptoms, including suicidal behavior in vulnerable individuals [20, 32].

Psychiatric disorders and comorbidities in early years influence academic functioning, and may subsequently lead to increased risk of dropping out of school [33] and receiving unemployment benefits or social insurance support [33]. In a population-based study in Central Norway, 17% was registered as being high school dropouts at age 24 [33], and more boys than girls were found to be non-completers in another Norwegian population-based survey [34]. According to World Health Organization, education and health are strongly linked [35]. School dropout was associated with poor mental health in a Danish population-based study [36], and school dropout involve heavy and enduring individual and social costs [37]. The link between suicidal symptoms, psychosocial and school function seems to be bidirectional; Adolescent self-harm or suicidal behavior are found to be associated with later mental health disorders and worse long-term functioning in young adulthood [38, 39]. According to a systematic review with meta-analysis of longitudinal studies, adolescents and young adults with school failure were at higher risk of suicide attempts [40]. There are many risk factors for school dropout [41], and reasons for leaving school vary widely [42]. Negative or stressful life events are found to be associated with intentions of and actual dropout [43], including conflicts with authorities for boys, and relational problems for girls [44]. High school students exposed to severe acute stressors are immediately vulnerable to dropping out [37].

The objective of the present study was to examine suicidality and functioning 3 years after referral to Child and Adolescent Mental Health Services. We aimed to assess psychosocial function, suicidal ideation, suicidal behavior, and school dropout, in the total sample and specified by psychiatric disorders, and furthermore to investigate associations with negative life events. We set out to specify analyses for girls and boys and explore gender differences. Hypotheses were that present suicidal symptoms and school dropout were associated with co-occurring exposure to negative life events, and furthermore, that frequencies differed between girls and boys, with girls having higher rates of suicidal symptoms and boys more school dropout.

## Method

### Study design

The Health Survey in Department of Children and Youth, Division of Mental Health Care, St. Olavs hospital, Trondheim University Hospital, Norway (St. Olav CAP Survey), is a prospective longitudinal cohort study of a defined clinical population assessed at two time points. Design and procedures are thoroughly described in former publications [45, 46]. At time point 1 (T_1_) (2009–2011), all patients aged 13–18 years who visited the Department of Children and Youth at least once over a 2-year study period, received oral and written invitations at their first attendance. The exclusion criteria were difficulties in answering the survey due to low cognitive function, visual impairments, insufficient language skills, or an unstable psychiatric state. Emergency patients were invited to take part once they entered a stable phase. The participants and their parents received standard application of services. They gave written informed consent to extract diagnostic data from clinical charts and respond to an electronic survey. At 3-year study follow-up (T_2_) (2012–2014), age 16–21 years, data were collected from the T_1_ enrolled sample and their parents, by an electronic survey and a diagnostic telephone interview performed by trained professionals.

### Participants

**Fig. 1:**
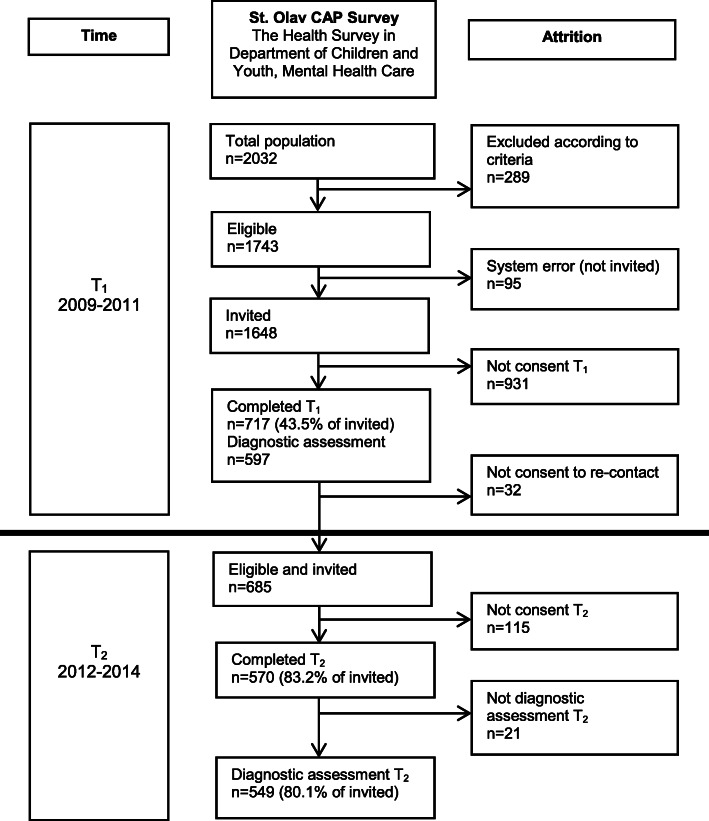
Flow-chart of the recruitment and attrition in the present study

In the T_1_ study period, 2032 adolescent patients had at least one attendance in the Department of Children and Youth [45, 46]. Figure 1 shows the participant flow in each stage of the survey. At T_1_, *n* = 717 participated (393 (54.8%) girls). At T_2_, all T_1_ participants who previously consented to further inquiry were invited (eligible *n* = 685), of whom 570 (83% of eligible) completed the follow-up questionnaire (324 (56.8%) girls), and 549 (80%) completed the diagnostic interview (308 (56.1%) girls).

### Participants vs. non-participants

To explore the representativeness of the study population at T_1_, anonymous information about the total clinical population was collected from annual reports from the Department of Children and Youth, 2009–2011, as previously published [45, 46]. All adolescents in the study period (*n* = 2032) minus those excluded (*n* = 289) were defined as reference population (*n* = 1743). The main reason for referral, age and gender were similar between participants (*n* = 717, 41.1%) and non-participants (*n* = 1026, 58.9%) (data not shown). Participants were 0.27 years older: Mean (SD) 15.7 (1.7) vs. 15.4 (2.0), and there were more girls among the participants: 393 (54.8%) vs. 509 (49.6%). Among those with participation at T_1_, there were 570 participants and 147 non-participants at T_2_. In depth attrition analyses are reported in a former publication [46]. Age and socioeconomic status were similar among participants and non-participants.

### Measures

**Psychiatric Diagnoses** at T_2_ were set using the semi-structured Schedule for Affective Disorders and Schizophrenia for School-Age Children (K-SADS) [47] according to the Diagnostic and Statistical Manual of Mental Disorders IV Text revision (DSM-IV-TR) [48]. Psychometric properties of the K-SADS, including reliability and validity, are found to be excellent [47], and the interview has previously been applied to populations in young adulthood [49, 50]. Adolescents were interviewed by telephone by trained interviewers, all with graduate degree in medicine or psychology and experience in child and adolescent psychiatric assessment. The interviewers met regularly with a supervisor, an experienced child and adolescent psychiatrist, to assure the quality and harmonization of the diagnostic assessment. All were blinded to T_1_ diagnoses. Inter-rater reliability in terms of negative agreement and positive agreement as recommended by van de Vet et al. [51]*,* was assessed using second ratings for 28 of the taped telephone interviews. Positive agreement varied from 0.615 to 1.000, and negative agreement varied from 0.884 to 1.000 [46]. The underlying contingency tables showing agreement are previously reported [46].

In the present study, disorders were grouped into the following categories, based on DSM-IV diagnoses at T_2_; Any psychiatric disorder, Anxiety disorders (DSM-codes 300, 308, 309), Mood disorders (DSM-codes 296, 300.4, 311), ADHD (DSM-code 314) and Other (DSM-codes 291, 292, 295, 298, 299, 301, 303, 304, 305, 307, 312, 313, 316). Due to few participants in some diagnostic groups, for example autism and eating disorders, and especially when examining suicidality and school dropout, we chose to merge children with these diagnoses into “other psychiatric disorders” for the purpose of this manuscript.

**The Children’s Global Assessment Scale (CGAS)** [52] was used to rate general psychosocial functioning on a scale from 1 (extremely impaired, needs constant supervision) to 100 (superior functioning), based on K-SADS interview. The CGAS is designed for children under 18 years, but was in this study used for all participants, also those above the age of 18 years. The inter-rater reliability for CGAS in terms of intraclass correlation coefficient (ICC) was 0.835, based on second ratings for 28 of the taped telephone interviews. Details are given in the Supplementary material (Table S1).

**Suicidal ideation or behavior** were measured at T_2_ by asking the following questions during K-SADS interview:

**Suicidal ideation**; “Sometimes children who get upset or feel bad think about dying or even killing themselves. Have you ever had such thoughts? How would you do it? Did you have a plan?” Assessed and scored as; 0; No information, 1; Not at all, 2; Infrequent or vague thoughts of suicide (e.g., less than once per month), or 3; Recurrent thoughts of suicide. As measure of **Suicidal ideation,** we used “infrequent or vague thoughts” (2) or “recurrent thoughts of suicide” (3), presently at T_2_.

**Suicidal acts or attempts**; “Have you actually tried to kill yourself? When? What did you do? Any other things? Did you really want to die? How close did you come to doing it? Was anybody in the room? In the apartment? Did you tell them in advance? How were you found? Did you really want to die? Did you ask for any help after you did it?” Assessed and scored as; 0; No information, 1; No attempt, 2; Preparations with no actual intent to die (e.g., held pills in hand) or planned attempt but did not follow through, 3; Self injurious behavior with any suicidal intent. There was one more assessment; “Ever attempted suicide”, scored as yes or no. In the present study, **Suicidal behavior** included “preparations or planned attempt” (2) or “self injurious behavior with any suicidal intent” (3), presently at T_2_, or yes to the question: “Ever attempted suicide”.

**School dropout** was self-reported at T_2_ based on answer “yes” to the following question: “Have you canceled your education (dropped out)?”

**Negative life events** were registered by self-report at both T_1_ and T_2_. At T_1_, the following questions were asked: “Have any of the following things happened to you?”; “That someone in your family has been seriously ill”, “Death of a loved one”, “A catastrophe (fire, avalanche, tidal wave, hurricane, etc.)”, “A serious accident (ex: a very serious car accident)”, “Been violently hurt (beaten or injured)”, “Seen others violently hurt”, “Been put in sexually uncomfortable/abusive situations by someone about your age”, “Been put in sexually uncomfortable/abusive situations by an adult”, “Been threatened or physically harassed by other students at school for a long time”, “Received painful or frightening treatment at the hospital while being treated for an illness or injury”. These items were also used in the Young-HUNT3 study (https://www.ntnu.edu/hunt/data/que).

At T_2_, the same questions were asked, and with a supplementary question: “Been seriously ill or injured”.

The answering opportunities were at T_1_; “No”, “Yes, last year” and “Yes, in my life”, and at T_2_; “No”, “Yes, last year” and “Yes, last three years”. In the present study, negative life events defined as “last 3 years” were events measured at T_2_ only, and negative life events defined as “ever” were measured at T_1_ or T_2_.

**Socioeconomic Status (SES)** was measured at T_1_ by the highest level of mothers’ education, divided into eight categories: 1) less than 9-year primary school; 2) completed 9-year primary school; 3) one or two years in high school; 4) completed high school; 5) completed high school and one-year education/training after high school; 6) academy/university for up to and including 4 years; 7) academy/university for 5 years or more; 8) academy/university including PhD.

### Statistical analyses

We compared proportions using the Newcombe hybrid score confidence intervals, as recommended by Fagerland, Lydersen and Laake [53], and the Pearson Chi squared test. Confidence intervals and tests for differences in psychosocial functioning between girls and boys were based on Student’s t-test for independent samples. We used binary linear regression with suicidal ideation, suicidal behavior or school dropout at T_2_ as dependent variables and negative life events reported at T_1_ and T_2_ as covariates, one at a time, to study their associations. The coefficients in binary linear regression represent risk differences. These regression analyses were carried out unadjusted and adjusted for SES as a possible confounder where relevant. Some estimates including suicidal behavior could not be computed when adjusting for SES due to non-convergence of the calculations. We report 95% confidence intervals (CI) where relevant, and two-sided *p*-values < 0.05 were considered statistically significant. Binary linear regression and the Newcombe CI were performed in Stata 16, and the other in SPSS 25.

## Results

### Suicidal measures and functioning

At T_2_, psychosocial functioning CGAS score was mean 71.3 (standard deviation 14.5) (Table 1). Girls had lower CGAS score than boys (mean 68.2 vs. 75.2, mean difference = − 7.0, CI (− 9.4 to − 4.7), *p* < 0.001). The frequency of suicidal ideation was 12.4%, girls 17.9% and boys 5.4% (risk difference; RD = 12.5%, CI (7.2 to 17.7), *p* < 0.001) (Table 1). Similar gender differences were found in suicidal behavior, were girls had the highest frequencies of suicidal attempts ever (25.0% vs. 9.5%, RD = 15.5%, CI (9.2 to 21.4), *p* < 0.001). School dropout was more frequent for girls than boys (22.5% vs. 13.2%, RD = 9.3%, CI (2.8 to 15.5), *p* = 0.006). Among those with a psychiatric disorder, suicidal ideation was higher among girls (24.8%), and suicidal behavior even higher (30.0%), RD for gender differences 18.0%, CI (10.8 to 24.7), *p* < 0.001, and 18.3%, CI (10.2 to 25.8), *p* < 0.001, respectively (Table 2). Specified by psychiatric disorder, girls had lower CGAS and higher frequencies of suicidal measures than boys in all diagnostic groups. The frequencies of suicidal ideation and behavior were highest in mood disorders and lowest in ADHD. For school dropout, gender difference was only found among patients with ADHD, with highest frequencies among girls (24.7% vs. 13.5%, RD = 11.3%, CI (0.3 to 22.3), *p* = 0.043).

**Table 1:** Clinical characteristics, psychosocial functioning, suicidal measures and school dropout at 3-year follow up

	Total			Girls			Boys			Girls versus Boys		
**Follow-up (T**_**2**_**)**	**n**	**Mean**	**(SD)**	**n**	**Mean**	**(SD)**	**n**	**Mean**	**(SD)**	**Mean difference**	**95% CI**^**a**^	***p*-value**^**a**^
Age (years)	570	18.7	(1.7)	324	19.0	(1.7)	246	18.3	(1.6)	0.7	(0.4 to 0.9)	< 0.001
SES	404	4.8	(1.7)	221	4.9	(1.7)	183	4.8	(1.8)	0.1	(−0.3 to 0.4)	0.714
CGAS	549	71.3	(14.5)	308	68.2	(15.5)	241	75.2	(12.0)	−7.0	(−9.4 to −4.7)	< 0.001
		**Proportion**	**(%)**		**Proportion**	**(%)**		**Proportion**	**(%)**	**Risk difference (%)**	**95% CI**^**b**^	***p*-value**^**c**^
Suicidal ideation^d^	549	68/548	(12.4)	308	55/307	(17.9)	241	13/241	(5.4)	12.5	(7.2 to 17.7)	< 0.001
Suicidal behavior	549	100/549	(18.2)	308	77/308	(25.0)	241	23/241	(9.5)	15.5	(9.2 to 21.4)	< 0.001
- Suicidal attempts presently	549	9/549	(1.6)	308	9/308	(2.9)	241	0/241	(0.0)	2.9	(0.8 to 5.5)	0.007
- Suicidal attempts ever	549	100/549	(18.2)	308	77/308	(25.0)	241	23/241	(9.5)	15.5	(9.2 to 21.4)	< 0.001
School dropout^e^	570	101/546	(18.5)	324	70^f^/311	(22.5)	246	31/235	(13.2)	9.3	(2.8 to 15.5)	0.006

***Note:*** *SES* Socioeconomic status*, SD* Standard Deviation*, CGAS* Children Global Assessment Scale (psychosocial functioning) (1–100, 1 = worst, 100 = best)

^*a*^ *Confidence intervals and tests for differences between girls and boys were based on Student’s t-test for independent samples*

^*b*^ *Newcombe hybrid score*

^*c*^ *Pearson Chi squared test*

^*d*^ *Suicidal ideation is defined as suicidal thoughts occasionally or often*

^*e*^ *School dropout includes patients answering yes to the question “Have you canceled your education (dropped out)?”*

^*f*^ *Of these, 6 had given childbirth*

**Table 2:** General psychosocial functioning, suicidal ideation or behavior and school dropout at 3-year follow up, specified by psychiatric disorders

	Total (***n*** = 549)		Girls (***n*** = 308)		Boys (***n*** = 241)		Girls versus Boys		
**Any psychiatric disorder**^**a**^	***n*** **= 385**		***n*** **= 223**		***n*** **= 162**				
	**Mean**	**(SD)**	**Mean**	**(SD)**	**Mean**	**(SD)**	**Mean difference**	**95% CI**^**d**^	***p*-value**^**d**^
CGAS	66.8	(14.0)	63.3	(14.4)	71.6	(12.0)	−8.3	(−10.9 to − 5.6)	< 0.001
	**n**	**(%)**	**n**	**(%)**	**n**	**(%)**	**RD (%)**	**95% CI of RD**^**e**^	***p*-value**^**f**^
Suicidal ideation^b^	66/384	(17.2)	55/222	(24.8)	11/162	(6.8)	18.0	(10.8 to 24.7)	< 0.001
Suicidal behavior^c^	86/385	(22.3)	67/223	(30.0)	19/162	(11.7)	18.3	(10.2 to 25.8)	< 0.001
School dropout	77/361	(21.3)	53/211	(25.1)	24/150	(16.0)	9.1	(0.5 to 17.1)	0.037
**Anxiety disorders**	***n*** **= 218**		***n*** **= 168**		***n*** **= 50**				
	**Mean**	**(SD)**	**Mean**	**(SD)**	**Mean**	**(SD)**	**Mean difference**	**95% CI of Difference**	***p*-value**
CGAS	61.6	(13.3)	60.5	(13.9)	65.2	(10.4)	−4.7	(−8.2 to −1.0)	0.012
	**n**	**(%)**	**n**	**(%)**	**n**	**(%)**	**RD (%)**	**95% CI of RD**	***p*-value**
Suicidal ideation	48/218	(22.0)	43/168	(25.6)	5/50	(10.0)	15.6	(2.7 to 24.7)	0.019
Suicidal behavior	64/218	(29.4)	55/168	(32.7)	9/50	(18.0)	14.7	(0.3 to 25.8)	0.045
School dropout	50/205	(24.4)	42/159	(26.4)	8/46	(17.4)	9.0	(−5.7 to 20.1)	0.209
**Mood disorders**	***n*** **= 98**		***n*** **= 80**		***n*** **= 18**				
	**Mean**	**(SD)**	**Mean**	**(SD)**	**Mean**	**(SD)**	**Mean difference**	**95% CI of Difference**	***p*-value**
CGAS	55.8	(11.3)	54.5	(11.4)	61.4	(9.6)	−6.9	(−12.7 to −1.2)	0.018
	**n**	**(%)**	**n**	**(%)**	**n**	**(%)**	**RD (%)**	**95% CI of RD**	***p*-value**
Suicidal ideation	42/98	(42.9)	38/80	(47.5)	4/18	(22.2)	25.3	(−0.0 to 42.4)	0.050
Suicidal behavior	39/98	(39.8)	36/80	(45.0)	3/18	(16.7)	28.3	(3.5 to 43.7)	0.026
School dropout	30/90	(33.3)	23/73	(31.5)	7/17	(41.2)	−9.7	(−34.4 to 13.0)	0.446
**ADHD**	***n*** **= 211**		***n*** **= 99**		***n*** **= 112**				
	**Mean**	**(SD)**	**Mean**	**(SD)**	**Mean**	**(SD)**	**Mean difference**	**95% CI of Difference**	***p*-value**
CGAS	69.2	(13.6)	65.9	(14.9)	72.1	(11.6)	−6.2	(−9.9 to −2.6)	0.001
	**n**	**(%)**	**n**	**(%)**	**n**	**(%)**	**RD (%)**	**95% CI of RD**	***p*-value**
Suicidal ideation	22/210	(10.5)	16/98	(16.3)	6/112	(5.4)	10.9	(2.6 to 20.0)	0.010
Suicidal behavior	41/211	(19.4)	27/99	(27.3)	14/112	(12.5)	14.8	(4.0 to 25.5)	0.007
School dropout	37/197	(18.8)	23/93	(24.7)	14/104	(13.5)	11.3	(0.3 to 22.3)	0.043
**Other psychiatric disorders**	***n*** **= 120**		***n*** **= 59**		***n*** **= 61**				
	**Mean**	**(SD)**	**Mean**	**(SD)**	**Mean**	**(SD)**	**Mean difference**	**95% CI of Difference**	***p*-value**
CGAS	63.2	(14.5)	57.7	(14.2)	68.4	(12.9)	−10.7	(−15.7 to −5.8)	< 0.001
	**n**	**(%)**	**n**	**(%)**	**n**	**(%)**	**RD (%)**	**95% CI of RD**	***p*-value**
Suicidal ideation	27/120	(22.5)	20/59	(33.9)	7/61	(11.5)	22.4	(7.5 to 36.4)	0.003
Suicidal behavior	31/120	(25.8)	23/59	(39.0)	8/61	(13.1)	25.9	(10.2 to 40.1)	0.001
School dropout	31/114	(27.2)	19/57	(33.3)	12/57	(21.0)	12.3	(−4.1 to 27.8)	0.141

**Note:** *SD* Standard Deviation*, CGAS* Children Global Assessment Scale (general psychosocial functioning) (1–100, 1 = worst, 100 = best)*, RD* Risk difference

^*a*^ *Psychiatric disorder includes both primary and additional diagnoses*

^*b*^ *Suicidal ideation is defined as suicidal thoughts occasionally or often*

^*c*^ *Suicidal behavior is defined as suicidal acts or attempts, presently at T*_*2*_ *or ever, also suicidal acts and attempts with suicidal thoughts*

^*d*^ *Confidence intervals and tests for differences between girls and boys were based on Student’s t-test for independent samples*

^*e*^ *Newcombe hybrid score*

^*f*^ *Pearson Chi squared test*

### Negative life events

Having serious illness of someone in family or death of a loved one, were the most common negative life events in this study (57.7% last 3 years and 85.7% ever), with higher frequencies among girls than boys only for the last 3 years (63.2% vs. 50.4%, RD = 12.8%, CI (4.5 to 20.8), *p* = 0.002) (Table 3). Having been seriously ill, injured or received painful or frightening treatment in hospital were more frequent among girls than boys both for the last 3 years and ever (26.5% vs. 16.5%, RD = 10.0%, CI (3.1 to 16.6), *p* = 0.005, and 38.0% vs. 27.0%, RD = 11.0%, CI (7.6 to 22.5), *p* < 0.001, respectively). Ever been exposed to a serious accident or catastrophe, were more frequent among girls (37.0% vs. 24.0%, RD = 13.0%, CI (5.4 to 20.4), *p* = 0.001). There were highly significant differences between girls and boys in having been put into sexually uncomfortable or abusive situations, both during the last 3 years and ever (23.5% vs. 2.9%, RD = 20.6%, CI (15.4 to 25.7), *p* < 0.001, and 44.4% vs. 7.9%, RD = 36.5%, CI (29.9 to 42.7), *p* < 0.001, respectively).

**Table 3:** Negative life events at 3-year follow up

Self-reported questionnaire (T_**1**_ and T_**2**_)^**a**^	Total (***n*** = 570)				Girls (***n*** = 324)				Boys (***n*** = 246)				Girls versus Boys			
	Last 3 years^**b**^		Ever^**b**^		Last 3 years		Ever		Last 3 years		Ever		Last 3 years		Ever	
	n	(%)	n	(%)	n	(%)	n	(%)	n	(%)	n	(%)	95% CI^**c**^	***p***^***d***^	95% CI	***p***
Serious illness of someone in family or death of a loved one	326/565	(57.7)	485/566	(85.7)	204/323	(63.2)	284/323	(87.9)	122/242	(50.4)	201/242	(83.1)	(4.5 to 20.8)	0.002	(−0.9 to 11.0)	0.080
Been seriously ill or injured, received painful or frightening treatment at hospital	126/566	(22.3)	178/566	(31.4)	86/324	(26.5)	123/324	(38.0)	40/242	(16.5)	55/242	(22.7)	(3.1 to 16.6)	0.005	(7.6, to 22.5)	< 0.001
Exposed to a serious accident or catastrophe	74/566	(13.1)	178/566	(31.4)	45/324	(13.9)	120/324	(37.0)	29/242	(12.0)	58/242	(24.0)	(−3.9 to 7.4)	0.506	(5.4 to 20.4)	0.001
Been threatened, physically harassed or violently hurt	124/566	(21.9)	262/566	(46.3)	77/324	(23.8)	160/324	(49.4)	47/242	(19.4)	101/242	(41.7)	(−2.6 to 11.0)	0.216	(−0.6 to 15.8)	0.088
Seen others violently hurt	131/566	(23.1)	241/566	(42.6)	65/324	(20.1)	140/324	(43.2)	66/242	(27.3)	101/242	(41.7)	(−14.4 to −0.2)	0.044	(−6.7 to 9.6)	0.726
Been put in sexually uncomfortable/abusive situations	83/566	(14.7)	163/566	(28.8)	76/324	(23.5)	144/324	(44.4)	7/242	(2.9)	19/242	(7.9)	(15.4 to 25.7)	< 0.001	(29.9 to 42.7)	< 0.001

***Note:*** ^*a*^ *Same questions at both T*_*1*_ *and T*_*2*_*, except for question “Been seriously ill”, which was only asked at T*_*2*_

^*b*^ *Negative life events defined as “last 3 years” were events measured at T*_*2*_ *only, and negative life events defined as “ever” were measured at T*_*1*_ *or T*_*2*_

^*c*^ *Newcombe hybrid score*

^*d*^ *Pearson Chi squared test*

### Associations

Binary linear regression with suicidal ideation as dependent variable and negative life events as covariates showed associations for several life events (Table 4). After adjustment for SES, the strongest associations were for been threatened, physically harassed or violently hurt (RD = 8.9%, CI (2.0 to 15.9), *p* = 0.012) and having been put into sexually uncomfortable or abusive situations (RD = 10.4%, CI (1.8 to 19.0), *p* = 0.018). Gender-specific analyses adjusted for SES, showed associations with having serious illness of someone in the family or death of a loved one, and being threatened, physically harassed or violently hurt for girls, but no associations were present for boys.

**Table 4:** Binary linear regression with suicidal ideation at 3-year follow up as dependent variable, and negative life events as covariates

Suicidal ideation^**a**^ at T_**2**_													
						Crude				Adjusted for SES			
Negative life events		No Neg. life event		Neg. life event		RD^**b**^	95% CI for RD			RD	95% CI for RD		
	n	n	(%)	n	(%)	%	Lower	Upper	***p*** value	%	Lower	Upper	***p*** value
**Total sample**	549												
Serious illness of someone in family or death of a loved one	535	3/73^c^	(4.1)	63/462^c^	(13.6)	9.5	4.0	15.1	0.001	8.1	0.9	15.2	0.027
Been seriously ill or injured, received painful or frightening treatment at hospital	536	39/367	(10.6)	27/169	(16.0)	5.3	−1.0	11.7	0.100	8.6	0.9	16.3	0.028
Exposed to a serious accident or catastrophe	536	40/365	(11.0)	26/171	(15.2)	4.2	−2.0	10.5	0.184	5.5	−2.2	13.3	0.160
Been threatened, physically harassed or violently hurt	536	26/287	(9.1)	40/249	(16.1)	7.0	1.4	12.7	0.015	8.9	2.0	15.9	0.012
Seen others violently hurt	536	32/306	(10.5)	34/230	(14.8)	4.3	−1.4	10.1	0.139	5.7	−1.4	12.7	0.115
Been put in sexually uncomfortable/abusive situations	536	36/383	(9.4)	30/153	(19.6)	10.2	3.3	17.2	0.004	10.4	1.8	19.0	0.018
SES	385					0.2^d^	−1.7	2.2	0.820				
**Girls**	308												
Serious illness of someone in family or death of a loved one	304	2/35	(5.7)	52/269	(19.3)	13.6	4.6	22.6	0.003	12.7	0.8	24.5	0.037
Been seriously ill or injured, received painful or frightening treatment at hospital	305	30/188	(16.0)	24/117	(20.5)	4.6	−4.5	13.6	0.322	8.4	−2.7	19.6	0.138
Exposed to a serious accident or catastrophe	305	31/192	(16.2)	23/113	(20.4)	4.2	−4.9	13.3	0.364	7.2	−4.3	18.7	0.221
Been threatened, physically harassed or violently hurt	305	21/153	(13.7)	33/152	(21.7)	8.0	−0.5	16.5	0.067	11.1	0.2	21.9	0.045
Seen others violently hurt	305	25/170	(14.7)	29/135	(21.5)	6.8	−2.0	15.5	0.129	9.1	−2.0	20.2	0.109
Been put in sexually uncomfortable/abusive situations	305	27/170	(15.9)	27/135	(20.0)	4.1	−4.6	12.8	0.354	6.1	−4.9	17.0	0.277
SES	210					−0.4	−3.6	2.8	0.806				
**Boys**	241												
Serious illness of someone in family or death of a loved one	231	1/38	(2.6)	11/193	(5.7)	3.1	−3.0	9.1	0.321	0.9	−6.8	8.6	0.821
Been seriously ill or injured, received painful or frightening treatment at hospital	231	9/179	(5.0)	3/52	(5.8)	0.7	−6.4	7.9	0.838	3.9	−4.9	12.7	0.387
Exposed to a serious accident or catastrophe	231	9/173	(5.2)	3/58	(5.2)	0.0	−6.6	6.6	0.993	3.0	−8.8	2.8	0.308
Been threatened, physically harassed or violently hurt	231	5/134	(3.7)	7/97	(7.2)	3.5	−2.6	9.6	0.261	3.6	−3.5	10.7	0.317
Seen others violently hurt	231	7/136	(5.2)	5/95	(5.3)	0.1	−5.7	6.0	0.969	0.8	− 5.9	7.4	0.820
Been put in sexually uncomfortable/abusive situations	231	9/213	(4.2)	3/18	(16.7)	12.4	−5.0	29.9	0.163	3.2	−11.0	17.4	0.659
SES	175					1.1	−0.1	2.4	0.072				

***Note*: Binary linear regression is based on paired data displayed in** Supplemental Material Table S2**.** SES Socioeconomic status

^*a*^ *Suicidal ideation includes suicidal thoughts occasionally or often*

^*b*^ *RD is risk difference, the difference between the proportions of patients with suicidal thoughts or behavior and negative life events compared with patients with suicidal thoughts or behavior without negative life event*

^*c*^ *The numbers in this table, for example 3/73 (4.1) and 63/462 (13.6), indicate that among the 73 patients with no negative life event, 3 had suicidal ideation at T*_*2*_*, and among the 462 patients with the negative life event, 63 had suicidal ideation at T2*

^*d*^ *The risk of having suicidal ideation increases with 0.2% per one unit increase in level of mothers education*

With suicidal behavior as dependent variable, adjusted associations were present for been seriously ill or injured (RD = 10.6%, CI (2.8 to 18.4), *p* = 0.008), exposure to a serious accident or catastrophe (RD = 10.1%, CI (1.9 to 18.3), *p* = 0.015), and been threatened, physically harassed or violently hurt (RD = 16.7%, CI (9.5 to 23.9), *p* < 0.001) (Table 5). Having seen others violently hurt was associated with suicidal behavior in girls only (RD = 14.6%, CI (3.4 to 25.8), *p* = 0.011). Some estimates could not be adjusted for SES due to non-convergence of the calculations. Thus, the association with having been put into sexually uncomfortable or abusive situations (RD = 21.8%, CI (13.6 to 29.9), *p* < 0.001) could not be adjusted for SES, neither could the corresponding association that was present only for girls (RD = 20.1%, CI (10.4 to 29.9), *p* < 0.001). Having been threatened, physically harassed or violently hurt was related to suicidal behavior for both girls (RD = 17.6%, CI (6.9 to 28.3), *p* = 0.001) and unadjusted for boys (RD = 12.0%, CI (3.8 to 20.2), *p* = 0.004). There was an association between SES and suicidal behavior (RD = − 2.3%, CI (− 4.4 to − 0.8), *p* = 0.005). Specified by diagnostic groups, associations with suicidal behavior were highly significant for Mood disorders and Anxiety disorders (*p* < 0.001) and the group Other disorders (*p* = 0.007), adjusted for SES (data not shown).

**Table 5:** Binary linear regression with suicidal behavior at 3-year follow up as dependent variable, and negative life events as covariates

Suicidal behavior^**a**^ at T_**2**_													
						Crude				Adjusted for SES			
Negative life events		No Neg. life event		Neg. life event		RD^**b**^	95% CI for RD			RD	95% CI for RD		
	n	n	(%)	n	(%)	%	Lower	Upper	***p*** value	%	Lower	Upper	***p*** value
**Total sample**	549												
Serious illness of someone in family or death of a loved one	536	8/73^c^	(11.0)	89/463^c^	(19.2)	8.3	−0.2	16.3	0.043	-^d^	–	–	–
Been seriously ill or injured, received painful or frightening treatment at hospital	537	50/368	(13.6)	48/169	(28.4)	14.8	7.2	22.5	< 0.001	10.6	2.8	18.4	0.008
Exposed to a serious accident or catastrophe	537	52/365	(14.2)	46/172	(26.7)	12.5	5.0	20.0	0.001	10.1	1.9	18.3	0.015
Been threatened, physically harassed or violently hurt	537	28/287	(9.8)	70/250	(28.0)	18.2	11.7	24.8	< 0.001	16.7	9.5	23.9	< 0.001
Seen others violently hurt	537	37/306	(12.1)	61/231	(26.4)	14.3	7.6	21.1	< 0.001	10.7	3.4	18.1	0.004
Been put in sexually uncomfortable/abusive situations	537	46/383	(12.0)	52/154	(33.8)	21.8	13.6	29.9	< 0.001	–	–	–	–
SES	386					−2.3^e^	−4.4	−0.8	0.005				
**Girls**	308												
Serious illness of someone in family or death of a loved one	305	7/35	(20.0)	68/270	(25.2)	5.2	−9.1	19.4	0.476	–	–	–	–
Been seriously ill or injured, received painful or frightening treatment at hospital	306	37/189	(19.6)	39/117	(33.3)	13.8	3.5	24.0	0.009	7.4	−3.5	18.3	0.184
Exposed to a serious accident or catastrophe	306	39/192	(20.3)	37/114	(32.5)	12.1	1.8	22.5	0.021	6.3	−5.1	17.6	0.280
Been threatened, physically harassed or violently hurt	306	22/153	(14.4)	54/153	(35.3)	20.9	11.5	30.3	< 0.001	17.6	6.9	28.3	0.001
Seen others violently hurt	306	27/170	(15.9)	49/136	(36.0)	20.1	10.4	29.9	< 0.001	14.6	3.4	25.8	0.011
Been put in sexually uncomfortable/abusive situations	306	27/170	(15.9)	49/136	(36.0)	20.1	10.4	29.9	< 0.001	–	–	–	–
SES	211					−3.6	−6.5	0.6	0.017				
**Boys**	241												
Serious illness of someone in family or death of a loved one	231	1/38	(2.6)	21/193	(10.9)	8.3	1.5	15.0	0.016	–	–	–	–
Been seriously ill or injured, received painful or frightening treatment at hospital	231	13/179	(7.3)	9/52	(17.3)	10.0	−0.9	21.0	0.073	–	–	–	–
Exposed to a serious accident or catastrophe	231	13/173	(7.5)	9/58	(15.5)	8.0	−2.1	18.1	0.122	11.1	−0.4	22.7	0.152
Been threatened, physically harassed or violently hurt	231	6/134	(4.5)	16/97	(16.5)	12.0	3.8	20.2	0.004	–	–	–	–
Seen others violently hurt	231	10/136	(7.4)	12/95	(12.6)	5.3	−2.7	13.3	0.196	5.1	−2.8	13.0	0.206
Been put in sexually uncomfortable/abusive situations	231	19/213	(8.9)	3/18	(16.7)	7.7	−9.9	25.4	0.390	15.7	−5.8	37.2	0.152
SES	175					−1.7	−3.4	−0.0	0.047				

*Note: Binary linear regression is based on paired data displayed in Supplemental Material Table S**3**. SES Socioeconomic status*

^*a*^ *Suicidal behavior includes suicidal acts or attempts, presently at T*_*2*_ *or ever, also suicidal acts and attempts with suicidal thoughts*

^*b*^ *RD is risk difference, the difference between the proportions of patients with suicidal thoughts or behavior and negative life events compared with patients with suicidal thoughts or behavior without negative life event*

^*c*^ *The numbers in this table, for example 3/73 (4.1) and 63/462 (13.6), indicate that among the 73 patients with no negative life event, 3 had suicidal ideation at T*_*2*_*, and among the 462 patients with the negative life event, 63 had suicidal ideation at T2*

^*d*^ *Estimates could not be computed due to non-convergence of the calculations*

^*e*^ *The risk of having suicidal behavior decreases with 2.3% per one unit increase in level of mothers education*

There were associations between school dropout and having seen others been violently hurt or been put in sexually uncomfortable/abusive situations, but after adjusting for SES, the associations only persisted for having seen others been violently hurt (RD = 10.8%, CI (2.9 to 18.8), *p* = 0.007), and only among girls (RD = 11.7%, CI (0.5 to 22.9), *p* = 0.041) (Table 6). Results were mainly unchanged when excluding those who gave childbirth (*n* = 6) (data not shown). An association found between suicidal behavior and school dropout was attenuated after adjustment for SES in the total sample (RD = 7.0%, CI (− 3.9 to 17.9), *p* = 0.209).

**Table 6:** Binary linear regression with school dropout at 3-year follow up as dependent variable, and negative life events as covariate

School dropout^**a**^ at T_**2**_													
						Crude				Adjusted for SES			
Negative life events		No Neg. life event		Neg. life event		RD^**b**^	95% CI for RD			RD	95% CI for RD		
	n	n	(%)	n	(%)	%	Lower	Upper	***p*** value	%	Lower	Upper	***p*** value
**Total sample**	570												
Serious illness of someone in family or death of a loved one	541	9/74^c^	(12.2)	91/467^c^	(19.5)	7.3	−1.0	15.6	0.083	5.8	−3.3	15.0	0.213
Been seriously ill or injured, received painful or frightening treatment at hospital	542	67/376	(17.8)	34/166	(20.5)	2.7	−4.6	9.9	0.472	3.3	−4.8	11.4	0.424
Exposed to a serious accident or catastrophe	542	65/371	(17.5)	36/171	(21.0)	3.5	−3.7	10.8	0.339	0.9	−7.1	8.9	0.830
Been threatened, physically harassed or violently hurt	542	43/289	(14.9)	58/253	(22.9)	8.0	1.4	14.7	0.017	6.2	−1.3	13.8	0.106
Seen others violently hurt	542	40/312	(12.8)	61/230	(26.5)	13.7	6.9	20.5	< 0.001	10.8	2.9	18.8	0.007
Been put in sexually uncomfortable/abusive situations	542	59/386	(15.3)	42/156	(26.9)	11.6	3.8	19.5	0.004	7.4	−1.4	16.3	0.100
SES	404					−1.1^d^	−3.2	1.1	0.319				
**Girls**	324												
Serious illness of someone in family or death of a loved one	310	5/35	(14.3)	64/275	(23.3)	9.0	−3.7	21.6	0.164	8.2	−5.7	22.2	0.247
Been seriously ill or injured, received painful or frightening treatment at hospital	311	42/195	(21.5)	28/116	(24.1)	2.6	−7.1	12.3	0.600	3.1	−8.1	14.3	0.588
Exposed to a serious accident or catastrophe	311	44/195	(22.6)	26/116	(22.4)	−0.2	−9.8	9.5	0.976	−1.7	−12.6	9.1	0.756
Been threatened, physically harassed or violently hurt	311	30/156	(19.2)	40/155	(25.8)	6.6	−2.7	15.8	0.165	2.2	−8.4	12.7	0.688
Seen others violently hurt	311	28/177	(15.8)	42/134	(31.3)	15.5	6.0	25.1	0.001	11.7	0.5	22.9	0.041
Been put in sexually uncomfortable/abusive situations	311	34/173	(19.7)	36/138	(26.1)	6.4	−3.0	15.9	0.181	3.7	−7.2	14.6	0.505
SES	221					−1.5	−4.8	1.7	0.351				
**Boys**	246												
Serious illness of someone in family or death of a loved one	231	4/39	(10.3)	27/192	(14.1)	3.8	−6.9	14.5	0.487	2.5	−9.2	14.2	0.674
Been seriously ill or injured, received painful or frightening treatment at hospital	231	25/181	(13.8)	6/50	(12.0)	−1.8	−12.1	8.5	0.731	0.6	−10.8	11.9	0.923
Exposed to a serious accident or catastrophe	231	21/176	(11.9)	10/55	(18.2)	6.3	−5.0	17.5	0.278	1.8	−10.1	13.6	0.770
Been threatened, physically harassed or violently hurt	231	13/133	(9.8)	18/98	(18.4)	8.6	−0.6	17.8	0.067	9.4	−1.5	20.4	0.092
Seen others violently hurt	231	12/135	(8.9)	19/96	(19.8)	10.9	1.6	20.2	0.022	9.3	−1.5	20.1	0.091
Been put in sexually uncomfortable/abusive situations	231	25/213	(11.7)	6/18	(33.3)	21.6	−0.7	43.8	0.057	10.2	−11.7	32.0	0.362
SES	183					−0.7	−3.3	2.0	0.621				

*Note: Binary linear regression is based on paired data displayed in Supplemental Material Table S**4**. SES Socioeconomic status*

^*a*^ *School dropout includes patients answering yes to the question “Have you canceled your education (dropped out)?”*

^*b*^ *RD is risk difference, the difference between the proportions of patients with school dropout and negative life events compared with patients with school dropout without negative life event*

^*c*^ *The numbers in this table, for example 9/74 (12.2) and 91/467 (19.5), indicate that among the 74 patients with no negative life event, 9 had school dropout at T*_*2*_*, and among the 467 patients with a negative life event, 91 had school dropout at T2*

^*d*^ *The risk of having school dropout decreases with 1.1% per one unit increase in level of mothers education*

## Discussion

This study is one of few surveys following a general clinical psychiatric population of adolescents who received standard clinical care, studying symptoms and function over time. Reassessed after 3 years, suicidal ideation and suicidal behavior were frequent, especially among girls, and across all subgroups of psychiatric disorders. Girls had lower psychosocial functioning than boys, and more school dropout. Associations were found between negative life events and suicidal ideation and behavior. Most marked were the associations between suicidal behavior and having been exposed to interpersonal violence for both girls and boys. For girls only, sexually uncomfortable or abusive situations were also related to suicidal ideation and suicidal behavior, as was having seen others been violently hurt. Furthermore, having serious illness of someone in family or death of a loved one were also associated with suicidal ideation for the total sample, and SES was linked to suicidal behavior for both girls and boys. School dropout was associated with having seen others be violently hurt among girls with ADHD.

In our sample assessed 3 years after referral for psychiatric disorders, the prevalence of suicidal ideation and behavior were similar to earlier research on clinical samples [7, 17]. The frequent occurrence of suicidal attempts may reflect that this is a follow up of former patients, with high rates of psychiatric disorders [46], and the results correspond well with earlier research describing that the majority of youth with suicidal behaviors have pre-existing mental disorders [5]. Still, the reasons for the high rates of suicidal ideation and behavior may be diverse, both depending on the persistence of psychiatric disorders [46], treatment given and the general vulnerability of the adolescents in this clinical population. There were large gender differences with girls having much higher rates than boys of both suicidal ideation and behavior, in line with earlier research [5], and especially described in the systematic review of 67 population-based longitudinal studies with focus on gender differences in suicidal behavior in adolescents and young adults [14]. In our study, almost one out of two girls with mood disorders had both suicidal ideation and suicidal behavior, whereas less than one out of four boys with mood disorders had the same symptoms. Boys with ADHD or other psychiatric disorders had the lowest frequencies of suicidal ideation or behavior. This follow up of former adolescent patients underscores the large gender differences and added risk for girls when it comes to suicidal symptoms.

Psychosocial function as measured by CGAS with values in the sub-normal range, indicated better functioning than expected in a clinical sample with frequent comorbidity. The inter-rater reliability was tested and shown to be good. CGAS was lower among girls than among boys, corresponding with earlier findings by Gårdvik et al. [46], showing that female participants had higher rates of psychiatric disorders and seemed to be more prone to develop co-occurring psychiatric disorders and a higher burden of disease. School dropout was also significantly higher among girls compared to boys, which may once again reflect a higher burden of disease among girls in this sample. Earlier research has showed that poor health, and especially mental health, has been significantly associated with dropout of school among adolescents, most marked for boys in higher education [33, 36]. Development and persistence of psychiatric disorders is prone to impact function in school and socialization, with possible long-term consequences [2]. It is therefore crucial to break the cycle at an early stage and hinder maintenance of problems.

The frequencies of experiencing negative life events are in accordance with earlier research reporting associations between psychiatric disorders and earlier negative life events or childhood adversities [54–56]. The most common experiences, in both genders, were serious illness of someone in the family or death of a loved one, reported for eight to nine out of ten adolescents. Almost half of the adolescents had been threatened, physically harassed or violently hurt, or seen others violently hurt, with no gender differences, whereas there were large gender differences in exposure to sexually uncomfortable or abusive situations. Less than one out of ten boys had such experiences, but almost one out of two girls were exposed. The results underline the importance of assessing negative life events among adolescents with psychiatric symptoms and disorders, in order to reveal any such risks, give proper treatment and if possible, prevent further traumatic events.

We examined possible associations between suicidal ideation at follow-up and negative life events. There was a significant association between suicidal ideation and having been threatened, physically harassed or violently hurt in the total sample, but in gender-specific analyses, the association was present only for girls. Suicidal ideation was also associated with exposure to sexually uncomfortable or abusive situations for the total sample, corresponding with earlier research described in the meta-analysis of 50 years of research by Franklin et al. [21]. Furthermore, we found that suicidal ideation was associated with having serious illness of someone in the family or death of a loved one, but only for girls. Losing a loved one by death may be a very stressful event for children and adolescents, and a systematic review and meta-analysis by Howarth et al., found that stressful life events increased the risk of both reported suicidal ideation and behavior [22].

Earlier studies have demonstrated associations between suicidal behavior in adolescents and experiences of negative life events, as described in the systematic review by Serafini et al. [20]. In our study, we found associations between suicidal behavior and all negative life events. There was a strong association between suicidal behavior and having been threatened, physically harassed or violently hurt, for the total sample and for both girls and boys, which is in line with previous research, as described in the meta-analysis by Castellví et al. [25]. Among adolescents, victimization by peers is highly prevalent and associated with increased risk of suicidal attempts, and the longer history of victimization, the greater risk [28]. For girls only, suicidal behavior was related to having seen others violently hurt, as reported in earlier research [27]. Suicidal behavior was furthermore associated with exposure to sexually uncomfortable or abusive situations for girls only. Sexual abuse or violence has been found to be strongly associated to suicide attempts and behavior [27], and contributing the most to suicide attempts in youths and young adults together with bullying [25]. Opposite to suicidal ideations, serious illness of someone in the family or death of a loved one, was associated with suicidal behavior for boys only. This association has been found in earlier research [26, 27], but not specified by gender. Having been seriously ill, injured or received painful or frightening treatment in hospital, was associated with suicidal behavior in the total sample, in line with the meta-analysis of 50 years of research [21]. In the WHO World Mental Health Surveys, a cross-national analysis of the associations between traumatic events and suicidal behavior were investigated, and accidents and disasters were associated with suicidal behavior [27], as also found in our study. A systematic review of population-based studies by Evans et al. [57], found that suicidal phenomena in adolescents were associated with female gender, mental health problems, negative life events and poor family functioning, corresponding well with our findings.

In our clinical sample, an association was found between suicidal behavior and SES, for both girls and boys. The risk of having suicidal behavior decreased for the total sample with 2.3% per unit increase in level of mothers’ education. This indicates that SES does have an effect on the presence of suicidal behavior at follow-up, and that higher maternal education may be a protective factor for development of these symptoms. A large national register-based study showed strong associations between SES and suicidal risk [58].

School dropout is related to many different risk factors. A meta-analytic review by Gubbels et al. [41] described 23 risk domains with significant overall effect on school dropout, where mental health problems of the child and adverse childhood experiences were two of these domains. In our sample, we found associations between school dropout and having seen others been violently hurt, among girls only. A recent systematic review and meta-analysis of longitudinal studies by Castellví et al. [40] showed that adolescents and young adults who had school failure were at higher risk of a suicide attempt. In our clinical sample, the association found between suicidal behavior and school dropout in the crude analysis, did not withstand adjustment for SES. Therefore, SES may be a confounding factor for associations between suicidal behavior and school dropout, but may as well reflect larger *p*-values due to lower number of participants with SES information, or that the attrition was not random, i.e. that those with SES information were not representative for the entire sample.

One strength of the present study is the inclusion of a large clinical sample receiving standard clinical care, assessed after 3 years with a high response rate from T_1_ to T_2_. Furthermore, suicidal ideation and behavior were assessed in-depth by clinicians during the diagnostic interview, and not based on self-report measures which involves the limitations of less accuracy in establishing psychopathology. Some limitations need to be considered. The attrition rate was high in the initial recruitment, and even though the T_1_ sample did not differ in age, gender or reason for referral compared to non-participants, we cannot exclude that this high attrition rate may have affected the results. Since there were more girls than boys among participants compared to non-participants in this study, we may have lost some of the boys with psychiatric disorders, suicidality symptoms and impaired function. Life events and school dropout were measured by self-report only. School dropout was reported by one question, and additional information would have strengthened the measure, either by using several questions or information from other sources, supplemented by asking about the subjective reasons for the respective school dropout. Family characteristics including unemployment and low socioeconomic status influence mental health in off-spring [59], and may have important influence on many of the negative life events measured in this study, and their associations with suicidal measures or school dropout. Using level of maternal education to indicate socioeconomic status may not enclose the complete concept of socioeconomics, and furthermore, this information was accessible for a reduced sample, which may not reflect the total study population. Also, the reduced sample resulted in reduced power in the association analyses. The fact that some estimates including suicidal behavior could not be computed due to non-convergence of the calculations, was a statistical limitation. Treatment plausibly impact the course of suicidal ideations or behavior, also both general psychosocial function and school dropout, and information on treatment measures would have strengthened this study.

### Clinical implications

The results of this study bring an important message to clinical practice. Even though clinicians know about increasing symptoms of suicidal ideation and behavior during adolescence, the self-reported high rates of suicidal attempts in this patient group should be an extra reminder, also the high rates of school dropout, especially among girls. The burden of exposure to negative life events must also be acknowledged. Comprehensive assessment of mental health problems should of course include important risk factors, and asking adolescent patients about suicidal ideation and behavior, experiences of negative life events and school functioning seems to be important, especially for female psychiatric patients, in order to reveal any such risks and prevent further traumatic events.

## Conclusion

In this clinical sample reassessed after 3 years, one out of four adolescent girls with a persisting psychiatric disorder had suicidal ideations, and one out of three had a previous history of suicidal behavior. Girls had lower psychosocial functioning and higher rates of school dropout and experiences of negative life events than boys. Negative life events, especially exposure to interpersonal violence, were associated with suicidal ideation, suicidal behavior or school dropout. The high frequency of suicidal symptoms, school dropout and experiences of negative life events, indicates a high burden of challenges in functioning. The results reinforce the need to include these symptoms and associated factors in an extensive follow-up of psychiatric disorders in this age group.

## Supplementary Information

**Additional file 1: Table S1** Design matrix for the re-scoring of telephone interviews. **Table S2** Suicidal ideation T_2_ and Negative life events. **Table S3** Suicidal behavior T_2_ and Negative life events. **Table S4** School dropout T_2_ and Negative life events

## Data Availability

The datasets analyzed during the current study are not publicly available due to privacy policy, but they are available from the corresponding author on reasonable request.

## Abbreviations

K-SADS: Kiddie SADS: Schedule for Affective Disorders and Schizophrenia for School-Age Children
DSM-IV-TR: Diagnostic and Statistical Manual of Mental Disorders IV Text revision
ADHD: Attention Deficit Hyperactivity Disorder
CGAS: Children’s Global Assessment Scale
SES: Socioeconomic status
RD: Risk difference
CI: Confidence interval
