# Supplementary material for: Suicidality, function and associated negative life events in an adolescent psychiatric population at 3-year follow-up
Source: BMC Psychiatry. 2021 Feb 18;21:109. doi: 10.1186/s12888-021-03100-w (PMC7893950; doi:10.1186/s12888-021-03100-w)
Supplement: Supplementary file 1 — Additional file 1: Table S1 Design matrix for the re-scoring of telephone interviews. Table S2 Suicidal ideation T2 and Negative life events. Table S3 Suicidal behavior T2 and Negative life events. Table S4 School dropout T2 and Negative life events [file 12888_2021_3100_MOESM1_ESM.docx]

**Supplementary Material**

**Suicidality, function and associated negative life events in an adolescent psychiatric population at 3-year follow-up.**

**Authors**

Kari Skulstad Gårdvik, MD^1,2^, Terje Torgersen, PhD^3,4^, Marite Rygg, PhD^5,6^, Stian Lydersen, PhD^1^ and Marit Sæbø Indredavik, PhD^5^

^1^ Regional Centre for Child and Youth Mental Health and Child Welfare, Department of Mental Health, Faculty of Medicine and Health Sciences, Norwegian University of Science and Technology, Trondheim, Norway

^2^ Department of Children and Youth, Division of Mental Health Care, St. Olavs hospital, Trondheim University Hospital, Trondheim, Norway

^3^ Orkdal District Psychiatric Centre, Division of Mental Health Care, St. Olavs hospital, Trondheim University Hospital, Trondheim, Norway

^4^ Department of Mental Health, Faculty of Medicine and Health Sciences, Norwegian University of Science and Technology, Trondheim, Norway

^5^ Department of Clinical and Molecular Medicine, Faculty of Medicine and Health Sciences, Norwegian University of Science and Technology, Trondheim, Norway

^6^ Department of Pediatrics, St. Olavs hospital, Trondheim University Hospital, Trondheim, Norway

**Corresponding author**

Kari Skulstad Gårdvik, MD, E-mail: [kari.s.gardvik@ntnu.no](mailto:kari.s.gardvik@ntnu.no) Telephone: (+47) 93688058

**Inter-rater reliability for the CGAS ratings:**

The IRR study was designed as follows: Seven of the interviewers were used as second opinion raters for taped telephone interviews. Each of these seven re-scored four interviews performed by four of the other six interviewers. Hence, the number of re-scored patients were 7x4=28. The design was constructed as shown in Table S1, to be as balanced as possible.

**Table S1 Design matrix for the re-scoring of telephone interviews**

|  |  | Second rater | | | | | | |  |
| --- | --- | --- | --- | --- | --- | --- | --- | --- | --- |
|  |  | B | C | E | D | F | G | A | Sum |
| First rater | B |  | 1 | 1 | 1 | 1 |  |  | 4 |
|  | C | 1 |  | 1 | 1 |  | 1 |  | 4 |
|  | E |  |  |  | 1 | 1 | 1 | 1 | 4 |
|  | D | 1 | 1 |  |  |  | 1 | 1 | 4 |
|  | F | 1 |  |  | 1 |  | 1 | 1 | 4 |
|  | G |  | 1 | 1 |  | 1 |  | 1 | 4 |
|  | A | 1 | 1 | 1 |  | 1 |  |  | 4 |
|  | Sum | 4 | 4 | 4 | 4 | 4 | 4 | 4 | 28 |

The CGAS score is a continuous variable. We used a mixed effect model with rating number (first or second opinion) as fixed effect, and with individual and rater as random factors, to estimate the intraclass correlation coefficient (ICC). In this design, the raters are crossed with individuals (not nested within individuals), as illustrated in (Rabe-Hesketh & Skrondal 2012) page 98 (1). ICC analyses were carried out using Stata 13.

In the mixed effect model, the average CGAS score for rating number 1 was 74.07. For rating 2, the average score was 1.43 (p=0.31) higher. There are 3 variance components (given the fixed effect of rating number):

Individual to be rated: 187.0117 = 13.675^2^

Rater: 9.789 = 3.129^2^

Residual: 27.120 = 5.208^2^

The total variance is

187.0117 + 9.789 + 27.120 = 223.9209 = 14.964^2^

It follows (Rabe-Hesketh & Skrondal 2012, page 437-441) that the between rater, within individual intraclass correlation estimate is

13.675^2^ / 14.964^2^ = 0.835

The variance between the raters was not statistically significant (Likelihood ratio test p=0.19). That is, there was no evidence that some raters tended to give systematically higher scores than others with respect to CGAS.

1. Rabe-Hesketh S, Skrondal A. Multilevel and longitudinal modeling using Stata: STATA press; 2008.

**Table S2 Suicidal ideation (SI) T_2_ and Negative life events (NLE)**

|  | **Negative life events** | **n** | **No SI and**  **No NLE** | **Yes SI and**  **No NLE** | **No SI and**  **Yes NLE** | **Yes SI and**  **Yes NLE** |
| --- | --- | --- | --- | --- | --- | --- |
| **Total**  **(n=549)** | Serious illness of someone in family or death of a loved one | 535 | 70 | 3 | 399 | 63 |
|  | Been seriously ill or injured, received painful or frightening treatment at hospital | 536 | 328 | 39 | 142 | 27 |
|  | Exposed to a serious accident or catastrophe | 536 | 325 | 40 | 145 | 26 |
|  | Been threatened, physically harassed or violently hurt | 536 | 261 | 26 | 209 | 40 |
|  | Seen others violently hurt | 536 | 274 | 32 | 196 | 34 |
|  | Been put in sexually uncomfortable/abusive situations | 536 | 347 | 36 | 123 | 30 |
| **Girls**  **(n=308)** | Serious illness of someone in family or death of a loved one | 304 | 33 | 2 | 217 | 52 |
|  | Been seriously ill or injured, received painful or frightening treatment at hospital | 305 | 158 | 30 | 93 | 24 |
|  | Exposed to a serious accident or catastrophe | 305 | 161 | 31 | 90 | 23 |
|  | Been threatened, physically harassed or violently hurt | 305 | 132 | 21 | 119 | 33 |
|  | Seen others violently hurt | 305 | 145 | 25 | 106 | 29 |
|  | Been put in sexually uncomfortable/abusive situations | 305 | 143 | 27 | 108 | 27 |
| **Boys**  **(n=241)** | Serious illness of someone in family or death of a loved one | 231 | 37 | 1 | 182 | 11 |
|  | Been seriously ill or injured, received painful or frightening treatment at hospital | 231 | 170 | 9 | 49 | 3 |
|  | Exposed to a serious accident or catastrophe | 231 | 164 | 9 | 55 | 3 |
|  | Been threatened, physically harassed or violently hurt | 231 | 129 | 5 | 90 | 7 |
|  | Seen others violently hurt | 231 | 129 | 7 | 90 | 5 |
|  | Been put in sexually uncomfortable/abusive situations | 231 | 204 | 9 | 15 | 3 |

**Table S3 Suicidal behavior (SB) T_2_ and Negative life events (NLE)**

|  | **Negative life events** | **n** | **No SB and**  **No NLE** | **Yes SB and**  **No NLE** | **No SB and**  **Yes NLE** | **Yes SB and**  **Yes NLE** |
| --- | --- | --- | --- | --- | --- | --- |
| **Total**  **(n=549)** | Serious illness of someone in family or death of a loved one | 536 | 65 | 8 | 374 | 89 |
|  | Been seriously ill or injured, received painful or frightening treatment at hospital | 537 | 318 | 50 | 121 | 48 |
|  | Exposed to a serious accident or catastrophe | 537 | 313 | 52 | 126 | 46 |
|  | Been threatened, physically harassed or violently hurt | 537 | 259 | 28 | 180 | 70 |
|  | Seen others violently hurt | 537 | 269 | 37 | 170 | 61 |
|  | Been put in sexually uncomfortable/abusive situations | 537 | 337 | 46 | 102 | 52 |
| **Girls**  **(n=308)** | Serious illness of someone in family or death of a loved one | 305 | 28 | 7 | 202 | 68 |
|  | Been seriously ill or injured, received painful or frightening treatment at hospital | 306 | 152 | 37 | 78 | 39 |
|  | Exposed to a serious accident or catastrophe | 306 | 153 | 39 | 77 | 37 |
|  | Been threatened, physically harassed or violently hurt | 306 | 131 | 22 | 99 | 54 |
|  | Seen others violently hurt | 306 | 143 | 27 | 87 | 49 |
|  | Been put in sexually uncomfortable/abusive situations | 306 | 143 | 27 | 87 | 49 |
| **Boys**  **(n=241)** | Serious illness of someone in family or death of a loved one | 231 | 37 | 1 | 172 | 21 |
|  | Been seriously ill or injured, received painful or frightening treatment at hospital | 231 | 166 | 13 | 43 | 9 |
|  | Exposed to a serious accident or catastrophe | 231 | 160 | 13 | 49 | 9 |
|  | Been threatened, physically harassed or violently hurt | 231 | 128 | 6 | 81 | 16 |
|  | Seen others violently hurt | 231 | 126 | 10 | 83 | 12 |
|  | Been put in sexually uncomfortable/abusive situations | 231 | 194 | 19 | 15 | 3 |

**Table S4 School dropout (SD) T_2_ and Negative life events (NLE)**

|  | **Negative life events** | **n** | **No SD and**  **No NLE** | **Yes SD and**  **No NLE** | **No SD and**  **Yes NLE** | **Yes SD and**  **Yes NLE** |
| --- | --- | --- | --- | --- | --- | --- |
| **Total**  **(n=570)** | Serious illness of someone in family or death of a loved one | 541 | 65 | 9 | 376 | 91 |
|  | Been seriously ill or injured, received painful or frightening treatment at hospital | 542 | 309 | 67 | 132 | 34 |
|  | Exposed to a serious accident or catastrophe | 542 | 306 | 65 | 135 | 36 |
|  | Been threatened, physically harassed or violently hurt | 542 | 246 | 43 | 195 | 58 |
|  | Seen others violently hurt | 542 | 272 | 40 | 169 | 61 |
|  | Been put in sexually uncomfortable/abusive situations | 542 | 327 | 59 | 114 | 42 |
| **Girls**  **(n=324)** | Serious illness of someone in family or death of a loved one | 310 | 30 | 5 | 211 | 64 |
|  | Been seriously ill or injured, received painful or frightening treatment at hospital | 311 | 153 | 42 | 88 | 28 |
|  | Exposed to a serious accident or catastrophe | 311 | 151 | 44 | 90 | 26 |
|  | Been threatened, physically harassed or violently hurt | 311 | 126 | 30 | 115 | 40 |
|  | Seen others violently hurt | 311 | 149 | 28 | 92 | 42 |
|  | Been put in sexually uncomfortable/abusive situations | 311 | 139 | 34 | 102 | 36 |
| **Boys**  **(n=246)** | Serious illness of someone in family or death of a loved one | 231 | 35 | 4 | 165 | 27 |
|  | Been seriously ill or injured, received painful or frightening treatment at hospital | 231 | 156 | 25 | 44 | 6 |
|  | Exposed to a serious accident or catastrophe | 231 | 155 | 21 | 45 | 10 |
|  | Been threatened, physically harassed or violently hurt | 231 | 120 | 13 | 80 | 18 |
|  | Seen others violently hurt | 231 | 123 | 12 | 77 | 19 |
|  | Been put in sexually uncomfortable/abusive situations | 231 | 188 | 25 | 12 | 6 |
